# Service Urgency for Children and Youth: The Development of an Algorithm to Identify Urgent and Emergent Service Users in Children’s Mental Health

**DOI:** 10.3390/ijerph23050603

**Published:** 2026-05-02

**Authors:** Shannon L. Stewart, Abigail Withers, Jeffrey W. Poss

**Affiliations:** 1 Faculty of Education, Western University, London, ON N6G 1G7, Canada; sstewa24@uwo.ca; 2 Division of Children’s Health and Therapeutics, Children’s Health Research Institute, Lawson Health Research Institute, Western University, London, ON N6G 1G7, Canada; 3 Child and Adolescent Psychiatry, Schulich School of Medicine and Dentistry, The University of Western Ontario, London, ON N6A 3K7, Canada; 4 School of Public Health Sciences, University of Waterloo, Waterloo, ON N2L 3G5, Canada; jwposs@uwaterloo.ca

**Keywords:** children’s mental health, service urgency, interRAI, triage, prioritization, decision-support algorithm

## Abstract

**Highlights:**

**Public health relevance—How does this work relate to a public health issue?**

- Long wait times and inconsistent triaging in children’s mental health services represent a critical public health challenge that can delay access to care for children with urgent needs.
- This study identifies service urgency by developing a standardized, data-driven triage algorithm using routinely collected clinical information.

**Public health significance—Why is this work of significance to public health?**

- The interRAI Children’s Algorithm for Mental Health and Psychiatric Services (ChAMhPS) demonstrates reliable discrimination of high-urgency cases to support equitable and timely access to care across Ontario, Canada.
- The algorithm reduces reliance on clinical judgement alone and strengthens consistency in prioritizing children and youth with complex and high-risk mental health needs.

**Public health implications—What are the key implications or messages for practitioners, policy makers and/or researchers in public health?**

- Integrating the ChAMhPS algorithm into care can support practitioners and agencies in making more systematic, transparent, and reliable triaging decisions at intake.
- Standardized urgency data can help to inform policy, resource allocation, and performance monitoring to improve access and outcomes in children’s mental health services.

**Abstract:**

Timely access to children’s mental health services depends on accurate identification of service urgency; however, triage practices in Ontario, Canada vary widely, contributing to prolonged wait times and inconsistent pathways to care. This study aimed to develop and validate an empirically based decision-support algorithm to support standardized triaging and prioritization in Ontario based children’s mental health agencies. Data were drawn from 17,564 children and youth aged 4–18 years assessed with the interRAI Child and Youth Mental Health Screener (ChYMH-S) as part of routine clinical practice. Interactive decision tree modelling was used to identify combinations of clinical indicators associated with high service urgency, with age-stratified models for children 7 years and younger, 8–11 years, and 12 years and older. The resulting interRAI Children’s Algorithm for Mental Health and Psychiatric Services (ChAMhPS) classified individuals into seven urgency levels. The algorithm demonstrated good discrimination for services required within seven days (c-statistic = 0.70) and for the urgency of a comprehensive assessment (c-statistic = 0.73), with stable performance across derivation and testing samples. Higher algorithm levels were associated with an increased likelihood of urgent assessment or service need. The ChAMhPS algorithm offers a standardized, empirically derived tool to support clinical decision-making and improve consistency in triage and prioritization of children and youth with urgent mental health needs.

## 1. Introduction

It is estimated that on a global level, one in eight children has a mental health disorder [1], and around 1.6 million Canadian children and youth have a mental health disorder [2]. These concerns are typically serious enough to cause distress and impairment in several areas of life, thus requiring treatment [1]. Mental health issues can be emergent in certain circumstances. For example, concerns related to suicidality require immediate attention to assess risk, monitor symptoms, and ensure safety [3]. Conversely, other issues may be less emergent but still require intervention, resulting in the need for accurate screening, triaging, and prioritization. Without proper identification and timely intervention, unidentified or untreated children’s mental health concerns can have significant and lifelong consequences [4]. For example, a recent study conducted by Chartier and colleagues [5] noted that those diagnosed with mental health disorders before the age of 18 are at a two to four times higher risk of not finishing high school, needing income assistance or social housing, involvement in the criminal justice system, attempting suicide, or dying by suicide. Several mental health disorders start in childhood or adolescence, and early intervention at this developmental stage can have short- and long-term benefits [6]. As part of early intervention, it is imperative that services are structured to support the needs of children and youth, specifically identifying the urgency of needs through triage and prioritization.

Many Canadian children and families are facing problematic wait times and difficulties accessing mental health services [2], and there is a call for appropriate and timely access to mental health services [5]. Once children access services, the screening and triage process is largely dependent on the agency and/or service provider. Some service providers may use standardized assessment tools to measure need, triage, and plan services; however, these are not consistent across agencies and service providers [7], with several agencies utilizing homegrown instruments with no known reliability or validity [8]. Further complicating triaging and prioritization is the variation in clinical skills and collaboration efforts amongst service providers and no standard definition for clinical decision-making methods [4,9] or care quality [10]. As a result, there is significant variability and an overall lack of standardization in the pathways to which children are triaged into mental health services in Ontario. These prolonged waitlists, fragmented service routes, and inconsistent triaging pathways can lead to children and families receiving inappropriate treatments, even after lengthy waiting periods.

Given that there are no recognized standardized assessments of urgency employed across the service sectors or care systems [11,12], it is necessary to employ efficient, culturally sensitive, and developmentally appropriate approaches to reduce waitlists and improve triaging and prioritization. While there are screening tools used in children’s mental health both within and outside of Canada (e.g., Strengths and Difficulties Scale [13], Warwick-Edinburgh Mental Wellbeing Scale [14], Patient Health Questionnaire 9 [15], Screen for Child Anxiety and Related Disorders [16]), many instruments in use address only singular problems (e.g., anxiety, depression), exhibit limited responsiveness, have cultural limitations, or experience ceiling effects in clinical samples. Additionally, certain instruments used within care settings demonstrate low predictive value, exclude trauma-related issues, overestimate certain disorders, exhibit poor cross-informant agreement, lack sensitivity, or do not provide coverage of key symptoms associated with service urgency, resulting in limited scope and utility [17,18,19,20,21,22,23].

Decisions about urgency and priority are often made inconsistently across the mental health service system [24] without identifying both the severity and pervasiveness of symptoms or disorders or the degree of urgency needed [25]. Moreover, when prioritizing need in child and youth mental health, the client’s chronological age and level of development should be taken into account, given that certain thoughts, emotions, and behaviours are adaptive and typical at one age but maladaptive and atypical at another [25].

Additionally, many triaging instruments currently in use are continuum-based conceptualizations, and these tools are limited in indicating mental health need because subscale symptoms are weighted equally (e.g., suicidal intent is weighted the same as low mood) [26]. In clinical practice, triaging and intervention decisions are often based on branching logic as opposed to linear combinations of predictors [27]. Consequently, employing a decision support model (i.e., algorithm) has been found to better predict service urgency because it takes into consideration interactions among predictors and can generate patterns of actionable items. Notably, recent research has found risk algorithms to be superior to screening across numerous performance metrics, thereby improving triaging and prioritization for those most in need [28].

Within the child and youth mental health context, both within Canada and abroad, there are few instruments that provide a stepped-assessment approach that is integrated into a comprehensive assessment-to-intervention system designed to support clinical decision making across service sectors utilizing a multidisciplinary, lifespan approach to care [29,30]. Clinician-rated approaches to assessments, unlike self-report, gather information from multiple sources (e.g., mental status examination, case records, and family interviews) to provide a more complete summary of the child or youth’s mental health [12]. Compared to clinician-rated screening instruments, self-report measures are often considered less effective for triaging because they are highly susceptible to individual bias and tend to over-triage, whereas clinician-rated scales provide a more objective, standardized assessment that can better determine the severity and urgency of a mental health need or condition [31].

Given the current challenges within the child and youth mental health service sector, this study sought to develop an algorithm from the interRAI Child and Youth suite of instruments (see Section 2.2) to identify the factors associated with high service urgency within the children’s mental health system in the province of Ontario, Canada. interRAI is an international not-for-profit consortium of expert researchers and clinicians from over 40 countries who develop instruments for vulnerable populations across the lifespan (please refer to www.interrai.org for more information about assessment tools). As there is no existing triaging and prioritizing system in the Ontario context, the Children’s Algorithm for Mental Health and Psychiatric Services (ChAMhPS) was developed to help service providers and agencies triage incoming cases and prioritize the most urgent cases appropriately. The primary aim of this article is to outline the development and validation efforts made in developing the ChAMhPS algorithm.

## 2. Materials and Methods

### 2.1. Sample

All data came from assessments of children and youth in Ontario, Canada. These participants were referred and screened for mental health services as part of usual clinical practice with the Child and Youth Mental Health Screener (ChYMH-S; see below for a description) [32,33]. Referrals were made to the agencies from a variety of sources, including family physicians, pediatricians, school personnel, parents, or other allied professionals. Contributing agencies use the ChYMH-S instrument voluntarily and represent a broad cross-section of services, ranging from provincial and community mandates, while spanning residential and outpatient programmes. For the purposes of this work, a representative sample was not a requirement; it required that the sample be sufficiently large and represent a wide variety of clinical presentations, which was the case.

Completed screening assessments were drawn from the period between March 2015 and November 2016. If an individual had more than one screening assessment, the first one was used for the 17,564 persons. There were 108 cases excluded due to missing or dubious responses to items.

In an effort to predict children and youth with the most urgent service needs, an expert committee was consulted to assist in the identification of independent variables to inform the specification of the dependent variable and provide input regarding the development of the algorithm. Experts (including child psychiatrists, psychologists, and allied health professionals) were asked to identify items or constructs associated with urgent and emergent service needs, reflecting client or family characteristics. Items that described the care provider or were under the direct control of the care provider were excluded to mitigate the possible risk of bias.

### 2.2. Measures

This study references two interRAI Child and Youth assessments within the suite of instruments. The interRAI Child and Youth Mental Health Screener (ChYMH-S) and the interRAI Child and Youth Mental Health Instrument (ChYMH). The interRAI ChYMH is a comprehensive assessment consisting of 400 items designed to understand a child’s physical, mental health, behavioural, and social-emotional functioning [34]. It can be used amongst children and youth 4–18 years old in mental health settings throughout their clinical care. It provides a variety of applications (e.g., scales/algorithms for outcome measurement, care planning protocols, case mix systems to support resource allocation and quality indicators) [29].

Conversely, the ChYMH-S is a brief standardized assessment intended to complement the full ChYMH for screening and intake purposes [29]. ChYMH-S items were selected from the ChYMH instrument for the purposes of supporting decision-making related to triaging, placement, and service urgency [29]. Extensive reliability and validity studies have been done on these instruments and their applications. For example, the ChYMH-S screener has strong inter-item reliability and good convergent validity with other child mental health assessment tools [32]. The psychometric properties, including reliability and validity, of these instruments and their scales have been previously published [32,33,35,36,37,38,39,40,41,42,43].

The ChYMH-S includes an item to record the assessor’s judgement regarding the urgency of the assessed individual to receive a comprehensive, face-to-face mental health assessment. Response options for this item are an ordinal list: same day, 1 to 3 days, 4 to 7 days, 8 to 14 days, more than 14 days, and not required. This was dichotomized to assessment urgency of 7 days or sooner and used as the dependent variable in the interactive decision tree modelling. This choice was guided by the distribution of the responses, whereby same day and 1 to 3 days were too rare (about 3% each) to model specifically, and the grouping of 7 days (or less) balanced the need to find the most urgent cases with the creation of a robust algorithm. In addition, a similar item records the assessor’s judgement regarding the urgency of receiving mental health services, using the same response options, and was an additional outcome variable.

### 2.3. Analysis

The analytic dataset of 17,564 was randomly divided into 70% derivation and 30% testing subsamples, as a precaution to manage over-fitting of the model. Independent variables were drawn from 87 clinical items recorded in the ChYMH-S.

Modelling was done using an interactive decision tree approach. SAS Enterprise Miner supports a classification modelling tool where the operator chooses branches of the tree using information on the power of all candidate independent variables, allowing many alternative constructs to be explored. The strength of decision trees, as opposed to regression, is that interactions among predictors can be more naturally discovered and implemented. The initial splits of the tree structure are especially important; age groups were used here (7 and under, 8 to 11, 12 and over) based on feedback from clinical experts and programme compatibilities, as well as providing strongly differentiated fundamental groups on which to build the remaining tree structures. Age was an intuitive first split, since the kinds of clinical needs young children present with and that drive urgency are likely to be distinct from those of teenage cases. Numerous options were explored, especially regarding final splits where available assessment counts become small. An expert committee’s clinical judgments, as well as explanatory power, were considered in selecting the final tree model. The reserved testing subsample was used to examine for evidence of over-fitting in the derivation.

There were 30 terminal nodes, which were subsequently grouped, aided by k-means clustering, into 7 levels for reasons of parsimony, with a score of 0 having the lowest likelihood of urgent assessment, and 6 having the highest likelihood.

The additional outcome (service urgency) was also examined. Because of a moderate correlation between the two measures (polychoric correlation of 0.67), we anticipated that the algorithm predicting assessment urgency would also be effective at predicting service urgency. SAS 9.4 and SAS Enterprise Miner 13.1 were used for the analysis.

## 3. Results

Of the 17,564 individuals, 49.1% were female, and the average age was 11.9 years (10.9 among males and 12.8 among females). The proportions of age 7 and under, 8 to 11, and 12 and older were 17%, 25.4%, and 57.5%, respectively. The cases came from 39 different service organizations. Additional descriptive characteristics, stratified by age, are provided in Table 1.

The proportion with assessment urgency rated as 7 days or less was 13.6% and was strongly related to age, the first split used in the decision tree logic. The rates were 7.1% among those 7 and under, 8.5% among those 8 to 11, and 17.9% among those 12 and over.

Using the selected algorithm logic, a comparison of distributions among the 30 branches, and overall, did not show evidence that chance associations had resulted in decision points that were unsupported in the testing cases. The goodness of fit (c-statistic) was just slightly weaker in the derivation sample: 0.72 compared to 0.73 in the derivation sample.

**Figure 1:**
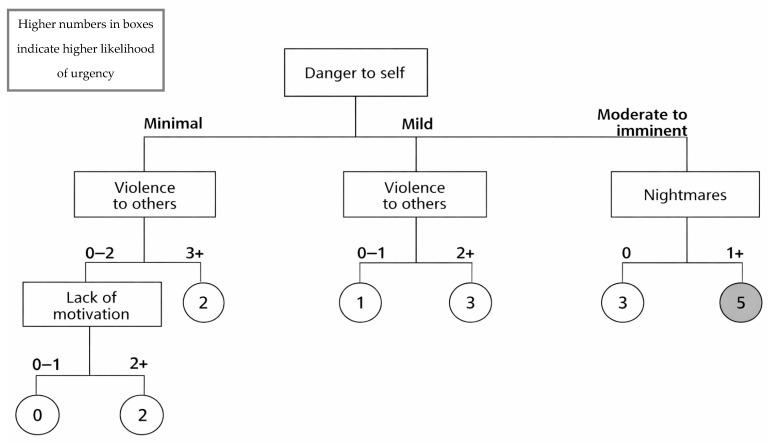
interRAI Children’s Algorithm for Mental Health and Psychiatric Services (ChAMhPS) for children ages 4–7.

**Figure 2:**
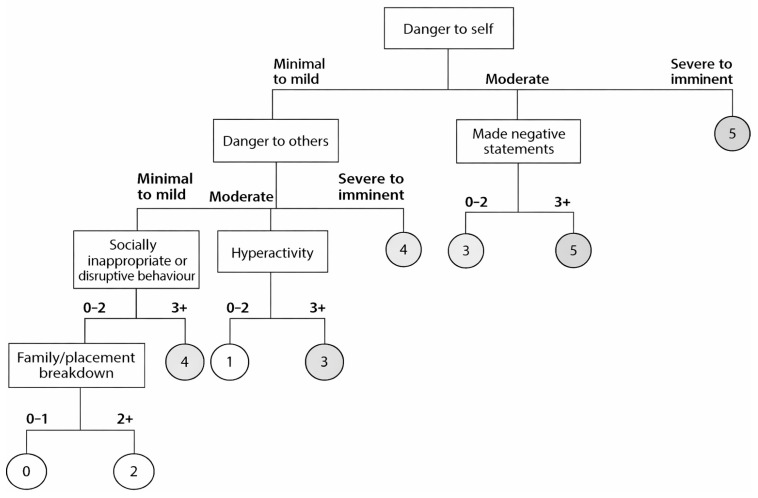
interRAI Children’s Algorithm for Mental Health and Psychiatric Services (ChAMhPS) for children ages 8–11.

**Figure 3:**
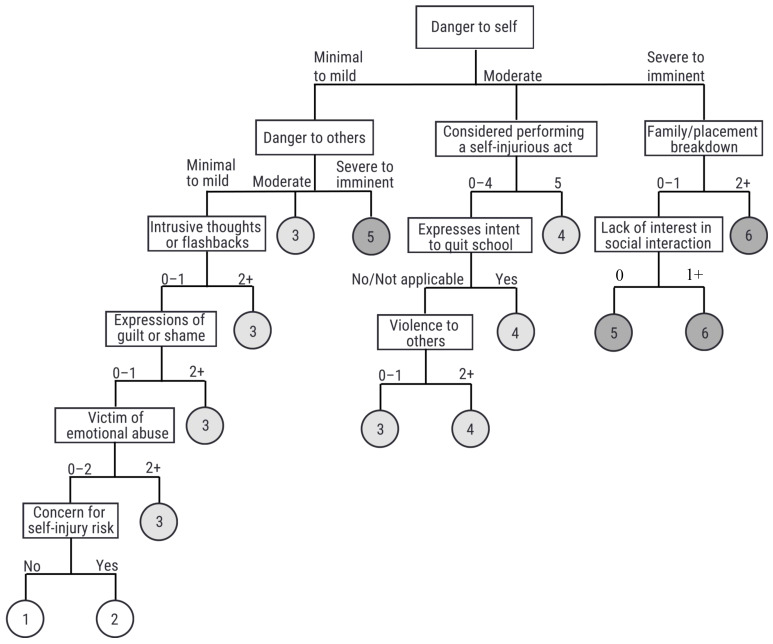
interRAI Children’s Algorithm for Mental Health and Psychiatric Services (ChAMhPS) for youth ages 12–18.

The logic diagram is depicted in Figure 1, Figure 2 and Figure 3, shown as a sub-tree for each of the age groups.

Table 2 summarizes the results. About 60% of cases were assigned the lowest two levels of urgency. The highest two levels of urgency were assigned 5.4% of cases.

Service required within 7 days shows overall strong associations with increasing levels of the service urgency algorithm. The c-statistic is just slightly lower than for assessment urgency (0.70 compared to 0.73).

In predicting the need for comprehensive assessment within 7 days, the algorithm also helps to predict the most urgent, those requiring same day attention. The odds ratio for the algorithm score of 6 (reference of 0) for same day assessment needs was 11.9 (confidence interval 8.1 to 17.5). However, some cases with same day assessment ratings will be classified as lower scores on the algorithm, underlining the need to treat this algorithm as a tool that can support clinical judgement, but not replace it.

## 4. Discussion

This paper presents the derivation of the interRAI Children’s Algorithm for Mental Health and Psychiatric Services (ChAMhPS), an empirically based decision-support tool that may be used to inform the need and urgency of timing for a comprehensive, face-to-face mental health assessment or service for children and youth between 4 and 18 years of age. The ChAMhPS algorithm is calculated based on several psychological, social, and behavioural factors for children and youth, with factors differing based on the child’s age. The algorithms provide a score ranging from 0 to 6, subdivided by age group (7 and under, 8 to 11, and 12 and older), based on urgency level for a full assessment or service need. The child or youth may fall into a given level via various pathways that represent different combinations of the criteria or risk factors (see Figure 1, Figure 2 and Figure 3) [33]. For those children and youth who receive a score of three and higher on the decision tree-based algorithm, further clinical assessment and more urgent or emergent mental health service is indicated.

Based on the findings, danger to self was one of the major predictors associated with emergent triaging and prioritization across all age ranges. Often, children and youth are seen in an acute crisis, requiring safety planning, supervision, and monitoring to ensure the child/youth’s wellbeing, which may also result in an inpatient stay for stabilization. Given that suicide is the second leading cause of death in youth [44], early identification is essential, and emergent action is required.

While certain criteria were consistent across all age ranges (e.g., danger to self or others), other criteria differed. Specifically, for children aged 7 years and younger, those children who received high scores on the ChAMhPS algorithm exhibited a combination of clinical indicators that included danger to themselves, nightmares, lack of motivation, and violence toward others in the last year.

Danger to self, combined with other risk factors such as behavioural problems, placed young children at heightened need for more urgent services. Notably, urgent and emergent pediatric referrals are most often related to aggression [45]. Specifically, children referred to mental health services for behaviour concerns, especially children exhibiting destructive and violent behaviour, are likely to receive service in a timely fashion [46,47]. Moreover, externalizing disorders (disruptive behaviours, aggression, and violence) have been found to be the most common reason for young children presenting to emergency departments [48]. At an early age, children who present with extreme behavioural difficulties often struggle with developmental issues (e.g., speech and communication issues), parent–child relationship difficulties, and underlying neurodevelopmental problems [49]. These behavioural problems often interfere with motivation, skill development, and executive functioning, leading to less optimal daily activity performance as well as poor cognitive and academic outcomes [50]. Based on extant literature, early presentations of behavioural difficulties are “red flags” often associated with the future development of psychiatric conditions such as autism spectrum disorder, attention-deficit hyperactivity disorder [48,49,51], intellectual disabilities, or health conditions [52].

Nightmares, when coupled with moderate to imminent danger to self, was indicative of the need for prompt prioritization. Research has suggested that nightmares are often associated with trauma experiences [53,54,55,56] as well as psychiatric issues [57,58]. Specifically, frequent nightmares in children are associated with anxiety, depression, hyperactivity, and even psychosis later in life [57,58].

For children between 8 and 11 years of age, the contributing factors related to high service urgency included being a danger to themselves and others, making negative statements, hyperactivity, socially inappropriate behaviour, and family breakdown. Similar to the younger age group, behavioural challenges continue to be a relevant factor involved in predicting service urgency for children between 8 and 11 years old. Unique to this age group, and as children age, peers become increasingly important [59], and children presenting with concerns in peer relationships, social skills, and social interactions are at an increased risk for mental health concerns, including anxiety and depression [60,61]. In the current study, it also indicated the need for a higher urgency when accessing clinical intervention and support.

Further complicating the service urgency of the 8- to 11-year-old age group is the presence of family challenges, including children at risk of being removed from their home (i.e., family/placement breakdown). This item reflects the level of risk associated with the child/youth experiencing a significant and potentially destabilizing life transition, such as separation from a primary caregiver or siblings, movement to a new home or placement, or disruption of established caregiving relationships. In many cases, risk of placement breakdown also indicates exposure to cumulative adversities, including family conflict, maltreatment, caregiver mental health difficulties, or prior system involvement. Children in foster care or those facing imminent placement changes are therefore navigating both psychosocial stressors and relational disruptions that elevate the risk of mental health concerns [62,63]. Children in our study identified as being at risk of these difficult life transitions were consequently in need of more urgent services. This finding is consistent with prior literature demonstrating that placement instability and caregiver disruption are strongly associated with emotional and behavioural difficulties, trauma-related symptoms, and service complexity [62,63]. Moreover, children in middle childhood are at a sensitive developmental period where disruptions in attachment and caregiving stability may have amplified effects on emotional regulation and social functioning [64,65].

In this middle childhood age group, making negative statements about one’s life, an indicator not observed among younger children, was associated with high service urgency. As children progress into middle childhood, they develop greater linguistic and emotional capacities, allowing them to verbalize internal distress and mood-related experiences more explicitly [66,67]. Children in this age group may begin articulating negative self or life evaluations, communicating their psychological distress verbally rather than solely through behaviour. The presence of these negative verbalizations represents their heightened emotional states and is associated with more urgent service needs.

As children age into adolescence, different factors become relevant as indications of mental health service urgency. For these children between 12 and 18 years old, the factors that contributed to high service urgency included the following: being a danger to themselves and others, violence to others, risk of self-injury, family breakdown, experiencing emotional abuse, lack of interest in social interaction, expressing intent to quit school, expressions of guilt or shame, and experiencing intrusive thoughts/flashbacks. For some factors, the mere presence of that factor was related to higher service urgency (e.g., lack of interest in social interaction, intent to quit school), whereas for other factors, recency (e.g., in the last 3 days) was related to higher service urgency.

Unique to this age group is the youth’s disengagement from social relationships and school activities, experiencing trauma or trauma symptoms, and the heightened risk of self-injury. During the adolescent developmental period, engagement in school and with peers is important to their development [59]. Therefore, those youth who are disengaged from school, peers, or social situations are likely to have an increased need for more urgent supports or services [61]. Previous research finds the proportion of students disengaged from school and requiring high-intensity mental health services increased with age, where older age groups of 12–18-year-olds were more likely to be disengaged in school compared to lower age groups [68]. Further complicating the clinical complexity and urgency for the adolescent age group in our study was the endorsement of trauma and trauma symptoms, and risk of self-harm [69], which are more commonly experienced in adolescent age groups [70]. Previous research using interRAI data demonstrates that co-occurring symptoms of self-harm ideation, depressive symptoms, and behavioural dysregulation are associated with increased risk of serious self-injury or violent behaviour and are therefore weighted heavily in algorithm scoring systems [71]. This aligns with broader evidence that combined internalizing and externalizing symptoms are related to emergency department mental presentations [72], thus indicating higher urgency for services or further assessment.

### 4.1. Use and Utility of the ChAMhPS

Based on these findings, the ChAMhPS algorithm is an empirically based decision-support tool for use in informing the urgency of children’s mental health needs. It is a good predictor of high urgency needs amongst children and youth, which can assist clinical staff when triaging and prioritizing clients, thereby providing a more informed, systematic, and standardized approach to service decisions. Once service providers complete the interRAI ChYMH-S assessment, they can view the ChAMhPS algorithm directly in the assessment software and interpret the results to assist with the determination of the child/youth’s urgency for service. If the ChAMhPS score is in the upper range, it is recommended that the clinical team consider how they can triage the young person as quickly as possible for further assessment or directly into services. The algorithm can therefore be used to assist with the prioritizing process to provide more efficient, effective access to care.

During the screening process, the ChAMhPS score, in conjunction with other clinical information, should inform service providers or the clinical team about the need and urgency for an in-depth, face-to-face assessment or service. Higher scores suggest more complex needs, which require a more comprehensive standardized assessment tool, such as the interRAI Child and Youth Mental Health Assessment (ChYMH) [34] which comprises 400 items, with detailed scales and algorithms that outline mental health risk, daily functioning, family needs [36], and case complexity [73]. Applications from the ChYMH include care planning, a case mix system to allocate resources appropriately [29,34], and quality indicators [10]. The interRAI ChYMH also consists of 30 Collaborative Action Plans (CAPs) [74] that can be used to identify children/youth who are at a heightened risk for specific care needs and areas for intervention, including attachment, education, social and peer relationships, and suicidality [74].

If an inconsistency occurs between the service provider’s view of case urgency and the algorithm score, it would be best that the higher urgency level be followed. It should be noted that not all possible combinations of potential variables or information contributing to urgency can be included in any assessment. Hence, the final determination about urgency level should be made by the clinical team; however, the ChAMhPS algorithm can inform the decision. Throughout the process, the child/youth and family members should be engaged in the decision-making process [33], ensuring a client-centred approach to care. 

In addition to using the ChAMhPS algorithm for individual-level planning, the standardized data can be used on an agency level or across agencies or jurisdictions for benchmarking by allowing comparisons of similar populations to inform policy, planning, and future practice [33]. Hence, individual mental health urgency levels can be used on a systems level to ensure consistent and standardized triaging and allocation of resources. The Office of the Auditor General of Ontario Special Report [75] highlights the absence of standardized assessment and data practices in children’s mental health systems, indicating variability across agencies at intake, in assessment tools, and wait-time definitions. This results in inconsistencies with interpreting need and delays in care, especially for children with severe, complex, or escalating service needs. As a result of these inconsistencies, it is difficult for agencies to distinguish priority levels. It has been recommended that the interRAI Child and Youth assessment-to-intervention system be mandated, given its scientific rigour to support consistency in assessment, risk identification, triaging, and prioritization across service providers [75,76]. Integrating the interRAI ChYMH-S, where the ChAMhPS algorithm is embedded, across children’s mental health services could provide a shared language of need and risk, improve comparability across agencies, and promote a standardized, evidence-based approach to service urgency that ensures children with the highest needs receive timely access to care.

### 4.2. Equity and Use of the ChAMhPS

In any clinical decision-making process, it is crucial to reduce the likelihood that socially marginalized groups will be disadvantaged to ensure fairness and equity [77]. Coupled with clinician input, empirically derived algorithms, such as the ChAMhPS, can provide a standardized case-finding methodology to assist in the determination of routine, urgent, and more emergent care [32,71,78]. Timely access to the most appropriate services based on severity level and recency of issues can be facilitated, thereby allowing more in-depth, comprehensive assessments to be reserved for children and youth with higher acuity levels and more complex co-occurring difficulties. Consequently, more extensive resources could then be aligned with those in greatest needs, resulting in more equitable care [30].

Additionally, the ChAMhPS algorithm can also be utilized to examine benchmarking around equity within children’s mental health. interRAI assessments are utilized across multiple nations, and have been found to ensure multi-cultural sensitivities, incorporating diversity and inclusion, which can assist with the appropriate allocation of resources and identify and provide targeted supports to children who require it the most. This scientifically rigorous approach to clinical decision making can support opportunities to support improved pathways to triaging and prioritization, supporting the role of context, developmental processes, and diversity, as well as interactive processes that shape a child’s mental health [30].

Within any system, reducing barriers and providing equitable services are key to improving individual, family, and societal outcomes. Triaging is a critical step to connecting children and their families to appropriate services, particularly when it is within a coordinated health care system. This is especially important given that there are several points of entry into the mental health service system for children and adolescents, including the education, community, health, youth justice, and child welfare sectors [79]. It is common for the aforementioned sectors to work independently of each other, resulting in a ‘silo’ effect, with limited information sharing. These ‘silos’ have contributed to a fragmented system of mental health services. As a result, it can be time-consuming and geographically challenging for children, adolescents, and parents to find the appropriate services [80,81].

Given the use and utility of interRAI assessment systems across various care settings, the use of an algorithm that can span multiple service sectors, disciplines, and cultures can be very helpful. For example, interRAI child and youth instruments are now being implemented across multiple nations and continents (e.g., Switzerland, United States, The Netherlands, India, Africa, Nepal, Sri Lanka) with diverse populations (e.g., multiple age ranges and developmental levels, races, culturally diverse sub-populations). Once the data is received from these nations, it will provide future opportunities to examine cross-national differences and examine the generalizability of the ChAMhPS algorithm across diverse groups across the globe.

### 4.3. Limitations

While this study has many strengths, it is not without limitations. The ChAMhPS algorithm was developed using data collected as part of routine clinical practice without access to information related to wait times. Future research should explore how the algorithm predicts reduced wait times longitudinally after implementation. It should be noted, however, that this is a complicated endeavour since there is no consensus on wait time measurement in the field of children’s mental health.

Since the algorithm is derived only from variables on the interRAI ChYMH-S, there were limitations regarding the inclusion of broader contextual factors that could influence service urgency, such as service capacity, wait times, and individual or family preferences. For example, although we consulted an expert committee, we did not consult those with lived experience regarding using an algorithm to help determine service urgency.

It should be noted that race and socioeconomic status could not be presented in this study due to reporting requirements from the Canadian Institute for Health Information. Ongoing interRAI research across multiple nations is currently underway, given that previous research on population health predictive models has suggested that certain models underestimate predicted probabilities for low-income families, underscoring the complexity of predicting urgency due to health literacy, provider bias, and other factors related to social determinants of health [77].

Finally, items chosen in the algorithm were selected based on clinical applicability, relevance, and statistical power, but do not represent all conceivable factors that may drive assessment and service use. Continued validation and longitudinal evaluation will be imperative to improve timely access to children’s mental health services.

## 5. Conclusions

The ChAMhPS algorithm provides agencies with an empirically based decision-support tool that can be used to inform individual and agency-level choices made related to allocating resources and prioritizing services for children and youth in need of mental health services. The ChAMhPS algorithm, as part of the ChYMH-S standardized assessment process, can be used at an individual level to support clinical decisions about client care and can be used with aggregated data to help inform policy planning and changes.

## Figures and Tables

**Table 1:** Sample description.

Age Group	4 to 7	8 to 11	12 to 18	All
*N*	2988	4469	10,107	17,564
Female	31.7%	36.5%	59.9%	49.1%
Primary language				
English	97.4%	96.7%	97.6%	97.3%
French	2.2%	2.9%	1.8%	2.1%
Other	0.4%	0.4%	0.6%	0.6%
Intellectual disability	2.6%	6.1%	5.9%	5.4%
Strong, supportive relationship with family	91.5%	90.9%	81.1%	85.3%
Trauma * in the last year	12.7%	12.7%	19.5%	16.6%
Depression Symptom Index 6+	25.3%	38.8%	45.0%	40.1%
Risk of self harm (RISSK scale 2+)	12.0%	25.0%	60.6%	43.3%
Risk of harm to others (RIO scale 2+)	60.1%	47.2%	26.0%	37.2%
Assessment urgency				
Not required	14.9%	15.6%	18.5%	17.1%
More than 14 days	60.9%	57.5%	38.8%	47.3%
8 to 14 days	17.2%	18.5%	24.8%	21.9%
4 to 7 days	4.4%	4.9%	10.0%	7.8%
1 to 3 days	1.5%	2.0%	3.7%	2.9%
Same day	1.1%	1.6%	4.1%	3.0%
Service urgency				
Not required	3.6%	2.9%	2.8%	3.0%
More than 14 days	70.0%	67.5%	48.2%	56.8%
8 to 14 days	17.5%	20.0%	30.5%	25.6%
4 to 7 days	5.8%	6.4%	11.6%	9.3%
1 to 3 days	1.7%	2.2%	3.9%	3.1%
Same day	1.4%	1.1%	3.1%	2.3%

* sexual assault/abuse, physical assault/abuse, emotional abuse, witnessed domestic violence.

**Table 2:** Proportions and assessment urgency and service urgency by algorithm level.

Urgency Algorithm Level	N (Proportion of Sample)	% Assessment Required Within 7 Days	Odds Ratio, 95% Confidence Interval	% Service Required Within 7 Days	Odds Ratio, 95% Confidence Interval
0 (lowest urgency)	5250 (29.9%)	4.9%	1.0 (Ref)	6.9%	1.0 (Ref)
1	5416 (30.8%)	8.7%	1.9 (1.6–2.2)	9.3%	1.4 (1.2–1.6)
2	1789 (10.2%)	15.5%	3.6 (3.0–4.3)	16.6%	2.7 (2.3–3.1)
3	3107 (17.7%)	19.2%	4.6 (4.0–5.4)	20.7%	3.5 (3.1–4)
4	1054 (6%)	29.3%	8.1 (6.8–9.8)	30.0%	5.7 (4.9–6.8)
5	563 (3.2%)	42.3%	14.3 (11.6–17.7)	37.7%	8.1 (6.6–9.9)
6 (highest urgency)	385 (2.2%)	65.5%	37.1 (29.1–47.4)	61.6%	21.5 (17.1–27.1)
ALL	17,564	13.6%	C = 0.73	14.6%	C = 0.70

## Data Availability

The datasets presented in this article are not readily available due to privacy and confidentiality of the participants and ethical restrictions.

## Abbreviations

The following abbreviations are used in this manuscript:

ChYMH	Child and Youth Mental Health Instrument
ChAMhPS	Children’s Algorithm for Mental Health and Psychiatric Services
ChYMH-S	Child and Youth Mental Health-Screener
